# Banded Gastric Bypass: Better Long-Term Results? A Cohort Study with Minimum 5-Year Follow-Up

**DOI:** 10.1007/s11695-016-2397-4

**Published:** 2016-10-07

**Authors:** Luc Lemmens

**Affiliations:** Abdominal Surgery, AZ Nikolaas, Campus Sint-Niklaas, Moerlandstraat 1, 9100 Sint-Niklaas, Belgium

**Keywords:** Banded gastric bypass, Silastic ring, BMI, Weight loss, %EWL, Weight regain

## Abstract

**Background:**

While gastric bypass has been the treatment of choice for morbid obesity, insufficient weight loss and even weight regain has been observed in a sub-group of patients. Dilatation of the pouch, pouch outlet, and proximal alimentary limb have been suggested to cause weight regain on the long term. The banded gastric bypass surgery has been introduced to overcome this problem.

**Methods:**

Four hundred thirty-two patients (*n* = 254, non-banded/*n* = 178, banded-GaBP Ring™) were followed-up for 5 years. Patients were evaluated for weight loss, % excess weight loss (%EWL), weight regain and BMI.

**Results:**

No significant differences between groups in the first year following surgery were observed in terms of weight loss and %EWL. %EWL at 5 years was as follows: non-banded 65.2 ± 20.0 %; banded 74.0 ± 15.1 %. At 5 years, the banded group showed more weight loss (non-banded 35.4 ± 12.5; banded 43.9 ± 11.9 kg, *P* < 0.0001); weight regain was significantly higher in the non-banded group (*P* < 0.0001). Only minor complications were reported; no signs of ring migration or slippage were reported.

**Conclusion:**

Although, following the first year after surgery, no differences in treatment groups were observed in terms of weight loss, 5 years following surgery, patients who received banded surgery maintained better weight loss and had less weight regain compared to the non-banded group. These results suggest that laparoscopic banded gastric bypass using a silastic ring was effective in maintaining weight loss on the long term, while the complication rate was low. The banded gastric bypass is regarded by us as the new gold standard.

## Introduction

The gastric bypass surgery has been the treatment of choice for morbid obesity [1, 2] and is by many considered the gold standard. An excess weight loss (EWL) between 60 and 70 % in the first year of gastric bypass surgery and few side effects and complications was reported in meta-analyses done by Garb et al. and Buchwald et al. [3, 4]. While many studies have shown excellent outcomes in terms of weight loss and reduction of co-morbidities on the short and the long term [4, 5], other studies have not. Some papers report a failure rate that increased to 25–40 % in patients followed longer than 3 years due to weight gain [6, 7], with superobese patients showing the greatest weight gain. Failure rates of up to 34.9 % have been reported following gastric bypass surgery [7]. While the cause of inadequate weight loss and weight regain is multifactorial, an increase in the gastric reservoir size due to dilatation of the pouch, stoma, and proximal small bowel is frequently suggested as one of the reasons. Patients are reported to eat as much as before the operation [7]. It is known that either a dilated pouch or a dilated pouch outlet can lead to a poor restriction, lack of satiety, and thus a regain of weight [8–10]. To prevent weight regain, a variation on the gastric bypass surgery was introduced: the banded gastric bypass. An initial randomized controlled trial was done to evaluate the weight loss and complication rate after banded gastric bypass using a silastic ring [11]. In this prospective cohort of 432 patients with a complete 5-year follow-up, we compared the banded with the non-banded gastric bypass and investigated the advantage of adding a ring to the gastric bypass to prevent weight regain.

## Materials and Methods

### Patients and Study Cohort

After performing more than 2200 biliopancreatic diversions (Scopinaro procedure) and some gastric bandings, we started with the gastric bypass in 2002. A total of 1288 gastric bypass operations was performed by a single surgeon between June 2002 and March 2015. Of these, 316 patients received a non-banded gastric bypass (non-banded) and 972 a banded gastric bypass (banded). Our study started as a part of an international multi-center randomized study comparing banded and non-banded gastric bypass patients. The study was approved by the Ethics Committee of the University of Freiburg (Germany) and by the ethics committee of our hospital. A written informed consent was obtained from all participants. Due to the high demand of patients at our center preferring a silastic ring around the pouch, we stopped the randomization and continued our study as a single center prospective study. After ending the RCT, patients made their choice between a banded or non-banded procedure after extensive information concerning pros and cons of a both procedures. The study described in this article presents a cohort of 432 consecutive patients treated at the AZ Nikolaas, Belgium (254 non-banded/178 banded) with a minimum follow-up of at least 5 years. Follow-up visits took place at 3 and 6 months; 1, 2, 3, 4, and 5 years after the operation.

### Operative Technique

All operations were done laparoscopically. A vertical gastric pouch of 6–7 cm was created on a 34 Fr oesogastric tube. The pouch length was the same in both procedures. A silastic ring (GaBP Ring™, Bariatec Corporation, Palos Verdes Peninsula, CA, USA) was placed around the pouch, 1–2 cm proximal of the hand sewn anastomosis. It was closed according to the manufacturer’s instructions and fixed with two resorbable sutures. Rings with a circumference of 6.5 cm (diameter of closed ring was 1.9 cm) were initially used in all patients. Later, 6.5 cm was used for females and 7.0 cm for males. For the placement of the ring, we used an atraumatic grasper to bring the ring through the lesser omentum even in between the vessels of the lesser curvature. Because the ring is very small, no further dissection was needed. We did not notice any bleeding or damage of the gastric wall by performing this maneuver. The placement of the ring added 1 or 2 min to the operation. It was essential that the calibration tube was inside the pouch at the moment of ring closure and that there was a 5 mm space between the ring and the pouch upon closure. A Roux-en-Y was constructed with a biliopancreatic limb of 50 till 75 cm and an alimentary limb of 100 cm in all procedures. The integrity of the anastomosis was tested by using an air bubble test. Most of the patients left the hospital on the second day after the operation.

### Outcomes

Patient weight and BMI were recorded prior to the operation and at each follow-up visit. Post-operative complications were recorded at each follow-up visit. As a standard for evaluation, weight loss (in kg) and weight change were reported as the percentage of excess weight loss (%EWL), which was calculated using the following formula: %Excess Weight Loss = Weight loss × 100 / Excess Weight. The ideal weight was derived from the Metropolitan Life tables as an average between the minimum and maximum ideal weight. The Excess Weight = Weight − Ideal Weight [12]. %BMI loss was calculated as %BMI loss at each time point compared to pre-surgical BMI. Weight regain in BMI points was measured as the lowest achieved BMI—the BMI at 5 years follow-up.

### Statistical Analyses

All statistical analyses were performed using SAS version 9.3. Mixed ANOVA was performed to investigate for treatment effects in weight loss, %EWL, and BMI. This model assumes that the treatment is a between-subject fixed effect (there are no other treatment options than banded and non-banded), and time is a within-subject random effect (0 through 60 months continuously). The results relied on the assumptions that there were no outliers, normality, homogeneity, and sphericity. Using SAS, fit diagnostics for weight loss, %EWL, and BMI showed no data points that were obviously different; the sample sizes were sufficiently large (>30 for each group) and the graphics displayed no evidence that the variance differed within or between groups. The mixed ANOVA was represented as a general linear mixed effects model, with indicator variables for treatment. A segmented regression with a breakpoint at 12 months was used to make models of the best linear fit. Therefore, tests for effects and effect estimates for data were performed between 3–12 and 24–60 months for weight loss and %EWL, while results for BMI data were for 0–12 and 24–60 months. Weight regain and %BMI loss were evaluated using a two-sample *t* test. A *P* value of <0.05 was considered significant. All numerical data was expressed as mean ± standard deviation.

## Results

### Patient Characteristics

Between June 2002 and August 2010, 432 patients underwent banded (*n* = 178) or non-banded gastric bypass (*n* = 254), of which 88.2 % patients completed the 5-year follow-up visit. Demographic characteristics are shown in Table 1. Mean pre-operative weight and BMI in the non-banded group was 113.4 ± 20.1 kg and 40.2 ± 4.7 kg/m^2^, respectively, and 118.2 ± 16.2 kg and 41.8 ± 4.2 kg/m^2^ in the banded group. Mean pre-operative % excess weight was 93.3 ± 21.5 % in the non-banded group and 101.9 ± 22.2 % in the banded group (Table 1).

**Table 1:** Baseline patient characteristics

	Non-banded	Banded
N	254	178
Lost to follow-up (%)	10.6	11.2
N completed 5 years follow-up	227	158
Mean age (years)	41.2 ± 12.5 (range 14–70)	38.6 ± 11.4 (range 17–72)
Male/female (%)	28/72	27.5/72.5
Mean pre-operative weight (kg)	113.4 ± 20.1 (range 79–259)	118.2 ± 16.2 (range 84–178)
Mean BMI (kg/m^2^)	40.2 ± 4.7 (range 30.1–59)	41.9 ± 4.2 (range 32.8–55)
Mean excess weight (kg)	55.0 ± 15.4 (range 26–159)	59.3 ± 12.4 (range 32.9–95.8)
Mean % excess weight	93.3 ± 21.5 (range 49–166)	101.9 ± 22.2 (range 55–174)

Data are presented as mean ± standard deviation

### Weight Loss and %EWL

**Fig. 1:**
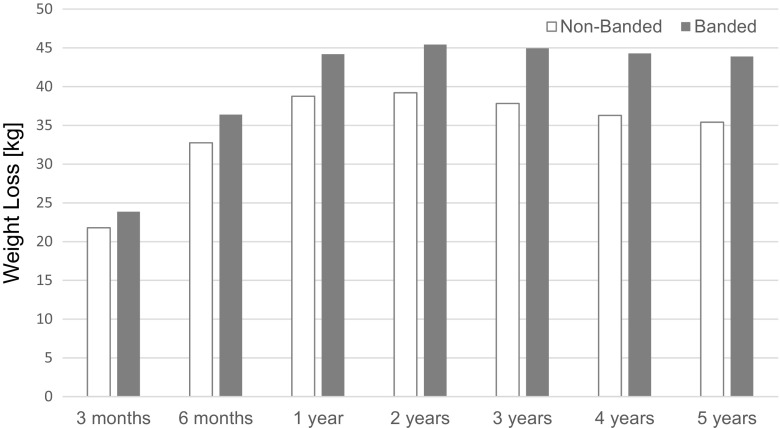
Evolution of weight loss. Data are displayed as mean weight loss in kg during the study period. Overall, these results show that over the first year, weight loss between banded and non-banded diverged quickly and over the next 4 years, while when weight loss was still divergent, there was a significant difference between the group’s average weight loss

The average weight loss and %EWL at 12 months was 38.8 ± 11.2 kg and 71.9 ± 18.2 %, resp., in the non-banded group and 44.2 ± 10.5 kg and 75.2 ± 13.9 % in the banded group, which was not significantly different between both groups over the 3–12-month period (*P* > 0.05), but a strong time (*P* < 0.0001) and time × treatment interaction effect (*P* < 0.0001, and *P* = 0.0465, resp., Table 2; Fig. 1) were observed.

**Table 2:** Mixed ANOVA-weight loss, % excess weight loss, and BMI

Parameter	Time point (months)	Non-banded	Banded	Significance	*P* value
Weight loss	3–12	38.8 ± 11.2	44.2 ± 10.5	T, T*tr	≤0.0001
	24–60	35.4 ± 12.5	43.9 ± 11.9	T, T*tr, tr	≤0.0001
%EWL	3–12	71.9 ± 18.2	75.2 ± 13.9	T, T*tr	≤0.0465
	24–60	65.2 ± 20.0	74.0 ± 15.1	T, T*tr	<0.0001
BMI	0–12	26.6 ± 4.5	26.3 ± 3.5	T, T*tr, tr	≤0.0002
	24–60	27.8 ± 4.9	26.4 ± 3.6	T, T*tr	<0.0001
Weight regain^a^	60	2.3 ± 2.3	1.2 ± 1.5	NA	<0.0001

Data are presented as mean ± standard deviation. Averages are provided at 12 months and at 60 months post-treatment. Data were analyzed using a mixed model ANOVA with time (T) indicating a time effect; time × treatment (T*tr) a significant interaction effect between time and treatment; treatment (tr) a significant treatment effect

^a^Weight regain was measured in the number of BMI points (lowest achieved BMI—BMI at 60 months)

**Fig. 2:**
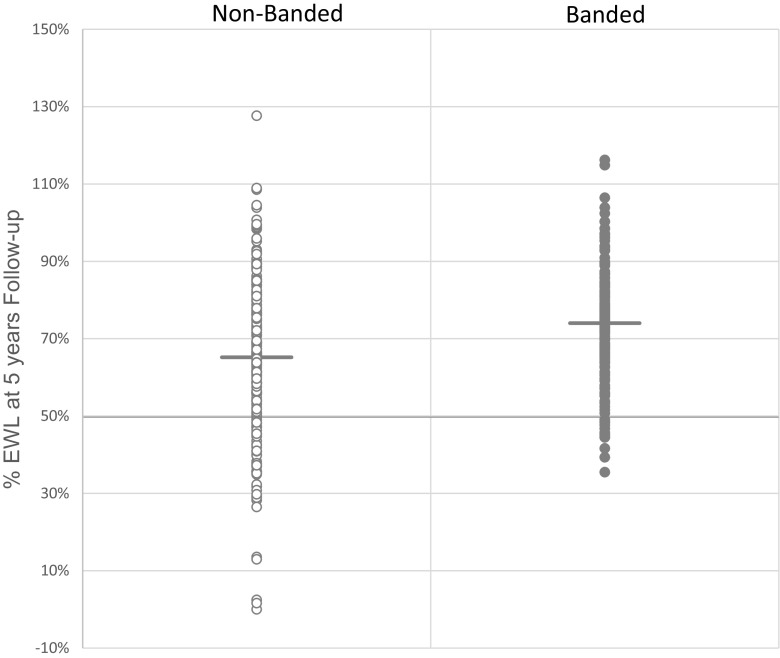
%EWL at 5 years follow-up. Data are displayed as the %EWL achieved by each patient at the 5 years follow-up visit in the non-banded and banded treated patients. *Lines* indicate the mean %EWL for each group (non-banded 65 %; banded 74 %). A *bold line* indicates the %EWL of 50 %: 79.3 % of the non-banded patients had %EWL >50 % (20.7 % had <50 %EWL); the banded patients had 92.4 % >50 %EWL (7.6 % had <50 %EWL)

The average weight loss and %EWL at 5 years was 35.4 ± 12.5 and 65.2 ± 20.0 %, resp., in the non-banded group compared to 43.9 ± 11.9 and 74.0 ± 15.1 %, resp., in the banded group. Over the 24–60-month period, a strong main treatment effect (*P* < 0.0001), a strong time, and a strong time × treatment interaction effect (*P* < 0.0001, and *P* = 0.0001, resp.) for weight loss were observed. These results indicate that over the first year, the weight loss between banded and non-banded diverged quickly and over the following 4 years, while when weight loss was still divergent, there was a significant difference between the group’s average weight loss with banded treated patients losing more weight. Additionally, there is strong evidence that the following 4 years saw an increase in %EWL in the banded group each month compared to the non-banded group. Figure 2 shows the distribution of %EWL for both groups at the 5 years follow-up visit. These results show that in the non-banded group 20.7 % of the patients had <50 %EWL at the 5 years follow-up, whereas 7.6 % of the banded treated patients had <50 %EWL.

### Body Mass Index

**Fig. 3:**
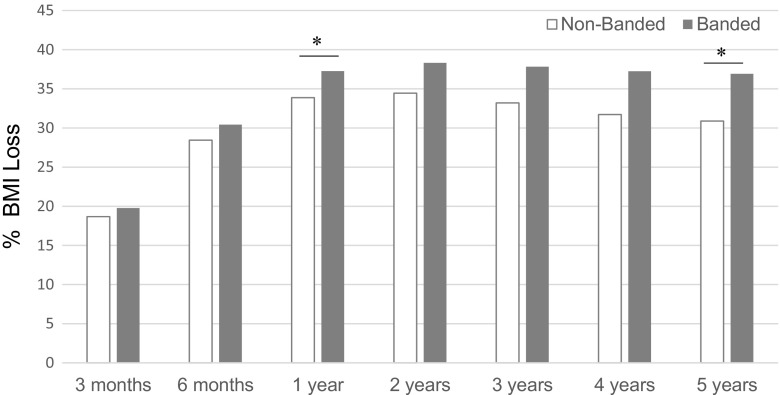
Evolution of %BMI Loss. Data are displayed as mean BMI during the study period. Overall, while the banded group had a higher BMI than the non-banded group over the first 12 months, it had a greater rate of decreasing. Significant more %BMI loss was observed for the banded group compared to the non-banded group at 1 and 5 years post-surgery (*Asterisk*)

The mean BMI at 12 months was decreased to 26.6 ± 4.5 kg/m^2^ in the non-banded group compared to 26.3 ± 3.5 kg/m^2^ in the banded group. The BMI results showed over 0–12 months, a strong main treatment effect (*P* = 0.0002), a strong time (*P* < 0.0001), and a strong time × treatment interaction effect (*P* = 0.0002; Table 2). %BMI loss at 1-year post-surgery was significantly higher in the banded group (37.3 %) when compared to the non-banded group (33.9 %; *P* = 0.0001; Fig. 3).

At 5 years, the mean BMI was 27.8 ± 4.9 kg/m^2^ in the non-banded group and 26.4 ± 3.6 kg/m^2^ in the banded group. Over the 24–60-month period, there was no main treatment effect (*P* > 0.05), a strong time effect (*P* < 0.0001), and evidence of a strong time × treatment interaction effect (*P* < 0.0001). Overall, these results show that while the BMI of the banded group over the first 12 months was higher than the non-banded group, it had a greater rate of decreasing, so much so that by year two through five, there was no evidence of a difference in BMI between the groups.

%BMI loss at 5 years post-surgery was significantly higher in the banded group (*P* < 0.0001; 30.9 %; banded 36.9 %) (Fig. 2).

### Weight Regain

**Fig. 4:**
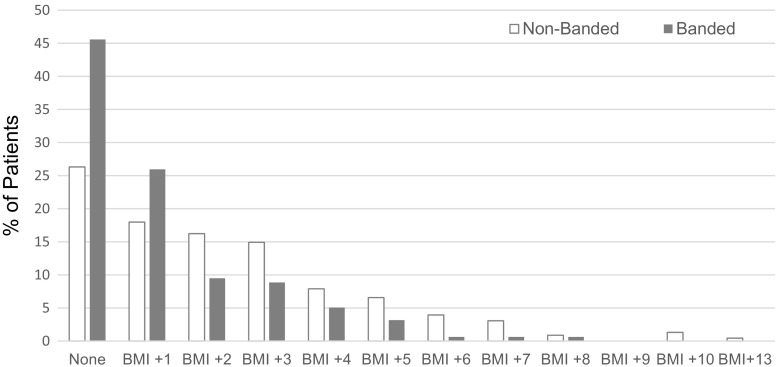
Weight regain at 5 years. Data are displayed as the % of patients who had an increase in BMI points at the 5-year follow-up period. Results show that the banded group had less weight gain compared to the non-banded group (*P* < 0.0001)

The weight regain results are shown in Fig. 4. The banded group had less weight regain at the 5 years follow-up visit compared to the non-banded group (*P* < 0.0001; Table 2). In the non-banded group, 26.5 % had no increase in BMI points compared to 45.6 % in the banded group, while 16.3 % of the patients in the non-banded group had an increase of >5 points BMI (*n* = 37) compared to 5.1 % of the patients in the banded group (*n* = 8). The most severe case of weight regain was observed in the non-banded group with an average increase of 13 BMI points.

### Complications

In the early post-operative follow-up, we noticed 4.4 % (19/432) early complications: leak (non-banded *n* = 2; banded *n* = 2), intra-abdominal bleeding (non-banded *n* = 5; banded *n* = 4), high intestinal bleeding (non-banded *n* = 2; banded *n* = 2), rhabdomyolysis (non-banded *n* = 1), and pulmonary embolism (non-banded *n* = 1). Late complications included internal hernia (non-banded *n* = 2), GE stenosis (non-banded *n* = 2; banded *n* = 1), and stomal ulcers (non-banded *n* = 3; banded *n* = 1). We could not find any difference in important early dumping between the two groups (non-banded 7 %; banded group 6 %). There were a few band-related problems: five patients complained about a functional stenosis at the level of the ring. In three patients, the ring was partially embedded into the fatty liver, and in two patients, there was an incompatibility with the ring. There was no difference in the eating behavior and late dysphagia in the first post-operative months between banded and non-banded patients. In the following years, more banded patients than non-banded patients feel some late dysphagia, sometimes even with vomiting when they eat too fast. The exact level of dysphagia is hard to quantify since these patients adapt to their eating pattern to their specific level of gastric restriction. Most of the patients do not complain about this because of the fear of weight regain in case of loss of restriction. In six patients, the ring was broken. These patients mentioned an increase in food intake with a subsequent weight regain and insisted themselves for a new ring. The first generation of the GaBP™ ring could break. One patient died, but this was not related to the bariatric surgery.

### Late Reoperations

There were 2.5 % non-specific late reoperations (*n* = 6 in the non-banded and *n* = 5 in the banded group; Table 3). A total reversal of the gastric bypass was performed in 1 patient because of untreatable dumping. In 5 patients (2.8 %), the ring was removed because of functional stenosis, and in 6 patients (3.4 %), the broken bands were replaced. Most importantly were the reoperations for weight regain. In the banded group, no reoperations for weight regain were required. In contrast, 14 patients in the non-banded group (5.1 %) received a ring of which 5 patients received a ring within the 5 years follow-up due to important weight regain. In 4 patients of the non-banded group (1.5 %), a distal gastric bypass was performed and in one patient a gastric bypass reversal was done.

**Table 3:** Late operations

	Non-banded (nr. pts)	Banded (nr. pts)
Laparoscopic adhesiolysis	1	1
Diagnostic laparoscopy (pain)	1	2
Intern hernia Petersen	2	0
Higher gastrectomy	2	0
Laparotomy perf. ulcer (elsewhere)	1	0
Laparoscopic closure ofgastric perforation after dilatation	0	2
Reversal of the gastric bypass (un-treatable dumping)	1	0
Band removal (functional stenosis)	NA	5 (2.8 %)
Band replacement (broken band)	NA	6 (3.4 %)

The banded gastric bypass ring

## Discussion

Despite the gain in interest of the sleeve gastrectomy, the gastric bypass is by many still considered as the gold standard for treating morbid obesity and is still the most commonly observed operation worldwide [13]. However, while gastric bypass is an effective method to quickly reduce weight, insufficient weight loss and even weight regain on the long-term have been shown for an important sub-group of patients. Results from this study demonstrate the quick weight loss during the first year following surgery showing a mean %EWL of 71.9 % in the non-banded group and 75.2 % in the banded group in the first year. These results indicate that gastric bypass was an effective method to quickly reduce weight and duplicate earlier findings showing a great reduction of weight in the initial year following surgery with %EWL ranging from 65 to 75 % [3, 14–16]. No statistically significant differences were observed in weight loss and %EWL between banded and non-banded patients during the 3–12 months following surgery, indicating that both treatments initially were equally effective in reducing weight. These good early results can be explained partially by the restriction and by the changes in the satiety gut hormones [27].

In contrast to the %EWL, weight loss, and weight regain, BMI showed a significant treatment effect after the first year, but not after 5 years. This could be explained by fact that the absolute BMI points of the banded group overall was higher than the non-banded group. However, the banded group had a greater reduction rate, so much so that at year 2–5, there was no evidence of a difference between BMI per group. The banded treated patients do show significant more %BMI loss at 1 and 5 years post-surgery when compared to the non-banded treated patients.

Of more interest is the difference between the treatment in terms of weight loss and weight regain during the 5-year time period. The evolution of weight loss shows a divergence between both groups with banded treated patients showing more weight loss between 1 and 5 years post-operatively (Table 2; Fig. 1). Weight regain in BMI points at 5 years shows pronounced differences with non-banded treated patients showing a greater weight regain (Table 2; Fig. 2). While in the non-banded group, only 26.3 % had no weight regain during the 5-year follow-up, and in 45.6 % of the banded group no weight regain was observed at 5 years. Most studies comparing banded versus non-banded bypass have confirmed these findings. Heneghan et al. [14] reported statistically superior weight loss in the banded group at 24 months, and in a sub-group analyses of this study, the authors show that superobese patients (BMI > 50) had even more benefit in terms of %EWL. Another study [17] showed that 10 years following surgery, the banded group achieved 81.7 %EWL compared to 62.3 %EWL in the non-banded group. Data from this study show (Fig. 2) that there are almost three times as many patients (20.7 %) in the non-banded group that end up below the 50 %EWL bracket after 5 years than in the banded group (7.6 %). This is a meaningful difference for patients in their choice of procedure. That banding helps in maintaining weight loss is further supported by the fact that patients experience weight gain when bands need to be removed for any reason [18].

Criticism could be made how the extra 15 % long-term excess weight loss can positively influence the metabolic effect of the procedure. It is known that metabolic disorders can reappear with weight regain. The scatter plot of Fig. 2 shows not only that the mean excess weight loss at 5 years is 15 % higher in the banded group but also that the standard deviation is much smaller in the banded than the non-banded group. This average 15 % difference is caused by the group of patients with an important weight regain or insufficient weight loss. Only 7 % of the banded group did not have the 50 % excess weight loss (Reinhold criteria) compared to 20 % in the non-banded group. In the banded group, those patients who did not reach the 50 % are also much closer to the 50 % mark than the non-banded group. Since the weight loss in the banded group is more sustainable, we expect this group to show improved metabolic results, but we have not measured this.

Weight regain after gastric bypass surgery has been attributed to enlargement of the gastric pouch and dilatation of the gastric pouch outlet which might lead to an enlarged gastric volume [19]. Already in 1980, MacArthur [20] described the dilatation of the pouch or pouch outlet to contribute to weight regain. More recent studies showed that pouch length, pouch volume, and stoma diameter correlated inversely with excess weight loss ±6 years after the primary surgery [19]. In a study by Yimcharoen et al. [9], a dilated gastrojejunostomy was seen in 59 % of the patients, a dilated pouch in 29 %, and a combination of both in 12 % of the patients ±7 years following bypass surgery. We are convinced that dilatation of the proximal jejunum, distal to the gastroenterostomy, plays an important role in the formation of the neo-stomach, leading to a complete loss of restriction. After a while, the gastric pouch becomes more flexible and (all) stomas will dilate. The small intestine will be the only cause of restriction [28] till it starts to dilate. This is well explained by the pain pattern of the patients: in the early post-operative period, patients will indicate the pain retrosternal because of the cardiac spasms. Later they mention the pain more in the epigastric region, which is explained by an intestinal spasm. Once the small intestine is dilated, the feeling of restriction is lost.

As pouch dilatation was observed, initially Linner [21] attempted to prevent this by reinforcing the gastroenterostomy anastomotic site with a silastic ring prosthesis. However, this resulted in a high incidence of band erosion and this procedure was abandoned. Fobi [22] reintroduced the ring and placed a silastic ring on a vertical pouch 2 cm above the gastroenterostomy rather than around the gastroenterostomy anastomosis [22, 23]. Ever since, several prosthetic devices have come to market, mostly silastic rings which are (laparoscopically) adjustable (MiniMizer®) or non-adjustable (GaBP Ring™) and may be placed around the pouch, proximal to the anastomosis. Other materials have been introduced, such as linea alba, fascia lata, meshes, porcine, and bovine grafts; however, silastic rings have been preferred by surgeons [24]. It has been suggested that a silicone band develops a pseudo-capsule which leads to less adhesion and is easier to remove than other materials, while other meshes have been shown to incorporate in scar tissue and are difficult to remove [25]. Other band-related complications have been reported such as infection, band erosion, migration, or stenosis.

Many surgeons show concerns regarding band migration or slippage of gastric rings. It has been well documented for adjustable gastric band that complications can occur till 15 years or later after the operation. In this study, 5 patients (2.8 %) in the banded group had their band removed due to functional stenosis, and in 6 patients (3.4 %), the band was replaced due to a broken band. In the initial GaBP™ ring, the teeth of the locking system could break. Despite the fact that newer GaBP™ ring have resolved this issue, we chose to change to the MiniMizer® ring for different reasons: this ring is adaptable from 6.5 to 8 cm, very flexible, easy to close, and did not break so far. This ring also has a soft needle, which ensures an easy closure of the ring and is very useful especially when using it for the banded sleeve. In case of incompatibility with the ring or functional stenosis, this ring can easily be opened by 0.5 cm or more. This can be done with 3 or 4 trocars of 5 mm on an out-patient basis. In this trial however, no signs of band erosion, or slippage were observed. Although not included in this study, we have performed more than 1.000-banded bypasses over more than 10 years and have seen only 1 intra-luminal migration and we were recently informed about 2 other migrations treated in another center. Next to that, we experienced 2 ring slippages in our own center. Neither of these complications were part of this study. The migration treated in our service was after a redo of a biliopancreatic diversion into a banded gastric bypass. In the patients with slippage, there was no fibrotic capsule formed around the ring. We therefore now fix the ring with 1 or 2 non-absorbable stitches. The low slippage rate of this ring can be explained by the small size of the ring which becomes surrounded by a thin fibrotic capsule already early after the operation in most of the patients. The low percentage of migration can be explained by the fact that the ring is placed loosely around the pouch, allowing for a 5 mm instrument to be put in between the pouch and the ring, where an adjustable gastric band is actively constricting the stomach every moment of the day. In the banded bypass, the ring is placed around a small pouch and will not cause compression of the stomach wall outside the meals. Only when a bigger food bolus is passing through there is a temporary compression, which causes some late dysphagia.

Compared to the observed complications in this trial, Heneghan reported similar late morbidity rates between banded and non-banded patients (10.4 versus 13.4 %), while 2.2 % band-related problems were reported [14]. Anecdotally, two patients who had their band removed (8 F silicone ring) and experienced weight gain. Bessler et al. [26] also reported no differences in complication rate between banded (26 %) and non-banded surgery (29.5 %). No band-related problems such as slippage or erosion were reported in this trial (Marlex band), and although the rate of wound infection was higher in the banded group, this was not significant. Emesis was found to be higher in banded patients compared to non-banded patients, though these were treated conservatively which was successful without requiring any intervention. Awad et al. [17] reported 3 band migrations (out of 260 banded surgeries) with a polytetrafluoroethylene (PFTE) ring in the beginning of their study. White et al. [5] reported a higher reoperation rate of 27 % of which 7 % were ring removals after silastic ring Roux-en-Y gastric bypass. In a meta-analyses by Buchwald et al. [4], who investigated 15 papers on banded bypass surgery, the authors reported a late complication rate of <6.0 %, a revision rate of <6.0 %, 2.3 % band erosion, 1.5 % band slippage, and 2.3 % band removal. Together with the results presented here, these studies suggest that although fear exists of band migration and band slippage, the actual rate of band-related problems were not as frequent and were resolved without severe sequelae.

## Conclusion

In summary, the results from this study show that laparoscopic gastric bypass was effective in quickly reducing weight following the first year after surgery, but 5 years following surgery, banded bypass surgery was more effective in reducing and maintaining weight compared to the non-banded group. More than 45 % of the patients in the banded group had no weight regain at all, compared to non-banded patients of which 26.5 % had no weight regain, but more than 16 % had an increase in BMI of more than five points 5 years following bypass surgery. Furthermore, the non-banded group had three times more patients that had less than 50 %EWL at 5 years follow-up. It is assumed that banding the pouch prevents pouch outlet dilatation and thus prevents weight regain on the long term and eventually revision surgery. The laparoscopic banded gastric bypass surgery was also found to be safe, with a similar complication rate between banded and non-banded surgery and a low incidence of band-related problems. While banded surgery seems more effective in maintaining weight loss on the long term compared to non-banded surgery, further prospective comparative long-term studies are required to confirm the safety of banded surgery and superiority over the non-banded surgery. The banded gastric bypass is the bariatric procedure of first choice in our service and could be considered the new gold standard.
